# The origins of limb lengthening and reconstruction surgery date back to 1521 when the first intervention ever reported in history was performed on St. Ignatius of Loyola

**DOI:** 10.1007/s00264-025-06591-4

**Published:** 2025-06-25

**Authors:** Filippo Vandenbulcke, Beth Lineham, Emiliano Malagoli, Alexander Kirienko

**Affiliations:** 1 IRCCS Humanitas Research Hospital, Via Manzoni 56, Rozzano - 20089, Milan, Italy; 2 Department of Biomedical Sciences, Humanitas University, Via Rita Levi Montalcini 4, Pieve Emanuele - 20072, Milan, Italy; 3 Trauma and Orthopaedics Department, Liverpool University Hospitals NHS Foundation Trust, Liverpool, UK

**Keywords:** History of orthopaedics, Limb lengthening, Reconstruction surgery, St. Ignatius of Loyola, Osteotomy, Non-union, Limb lengthening reconstruction surgery

## Abstract

**Purpose:**

To explore the historical case of Saint Ignatius of Loyola’s leg injury and subsequent surgical interventions as a potential early instance of limb lengthening and reconstruction surgery.

**Methods:**

A detailed analysis of “A Pilgrim’s Journey” (Ignatius of Loyola’s autobiography) was conducted, focusing on orthopaedic descriptions of his injury and treatments.

**Results:**

In 1521, Íñigo López de Loyola sustained a severe, comminuted open fracture of the tibia due to a cannonball wound during the siege of Pamplona. Initial attempts at reduction were unsuccessful, leading to a non-union with significant deformity and shortening. He underwent a revision surgery, a procedure described as “carnage” and endured without a single lament. Although the fracture eventually united, residual shortening and a prominent bone deformity persisted. Unwilling to accept this disfigurement for social reasons, Ignatius requested a second, highly painful osteotomy to remove the protruding bone followed by continuous traction for “days and days of martyrdom” for progressive lengthening. Crucially, after these arduous treatments, Ignatius was able to walk and even ride a horse again. The only significant residual symptom was swelling in his leg by evening.

**Conclusion:**

St. Ignatius of Loyola’s case provides a compelling historical account of complex orthopaedic challenges in the early 16th century. The documented surgeries represent remarkably early attempts at managing non-union, deformity, and potentially achieving limb lengthening, predating modern reconstructive techniques by centuries. This historical narrative offers valuable insights into the nascent stages of orthopaedic surgery and highlights how a physical ordeal can profoundly shape one’s life path.

## Introduction

The history of orthopaedic surgery is rich with examples of innovative solutions to complex musculoskeletal problems, often predating formal scientific understanding. While modern limb lengthening and reconstruction surgery has evolved significantly with advanced techniques and technologies, its conceptual roots can be traced to surprisingly early accounts of managing severe bone deformities. This article examines one such extraordinary historical case: the multifaceted surgical journey of Íñigo López de Loyola, later known as Saint Ignatius of Loyola, following a devastating battlefield injury in the 16th century. His “Pilgrim’s Journey” offers a unique, albeit non-medical, perspective on a series of orthopaedic interventions that bear striking resemblances to the principles of limb reconstruction [1].

It is generally recognized that modern limb lengthening techniques trace their origins to the early 20th century [2]. Alessandro Codivilla, often referred to as the “Father of Modern-Day Leg Lengthening,” was a pioneer in this field. In 1905, he described using skeletal traction for acute femoral lengthening after osteotomy [3]. His protégé, Vittorio Putti, further advanced these concepts, publishing his philosophy of femoral lengthening and an innovative device called an “osteon” in 1921. These foundational works mark the beginning of what is widely considered the modern era of limb lengthening [4]. However, it was the groundbreaking work of Gavriil Ilizarov in the mid-20th century, with his development of distraction osteogenesis and the external circular fixator, that truly revolutionized the field, allowing for precise, controlled bone regeneration and complex limb deformity correction [5, 6]. The case of St. Ignatius of Loyola suggests that the conceptual groundwork for such interventions may have been laid much earlier, through empirical, albeit painful, surgical practices [7].

### The Pamplona injury and initial management

**Fig. 1:**
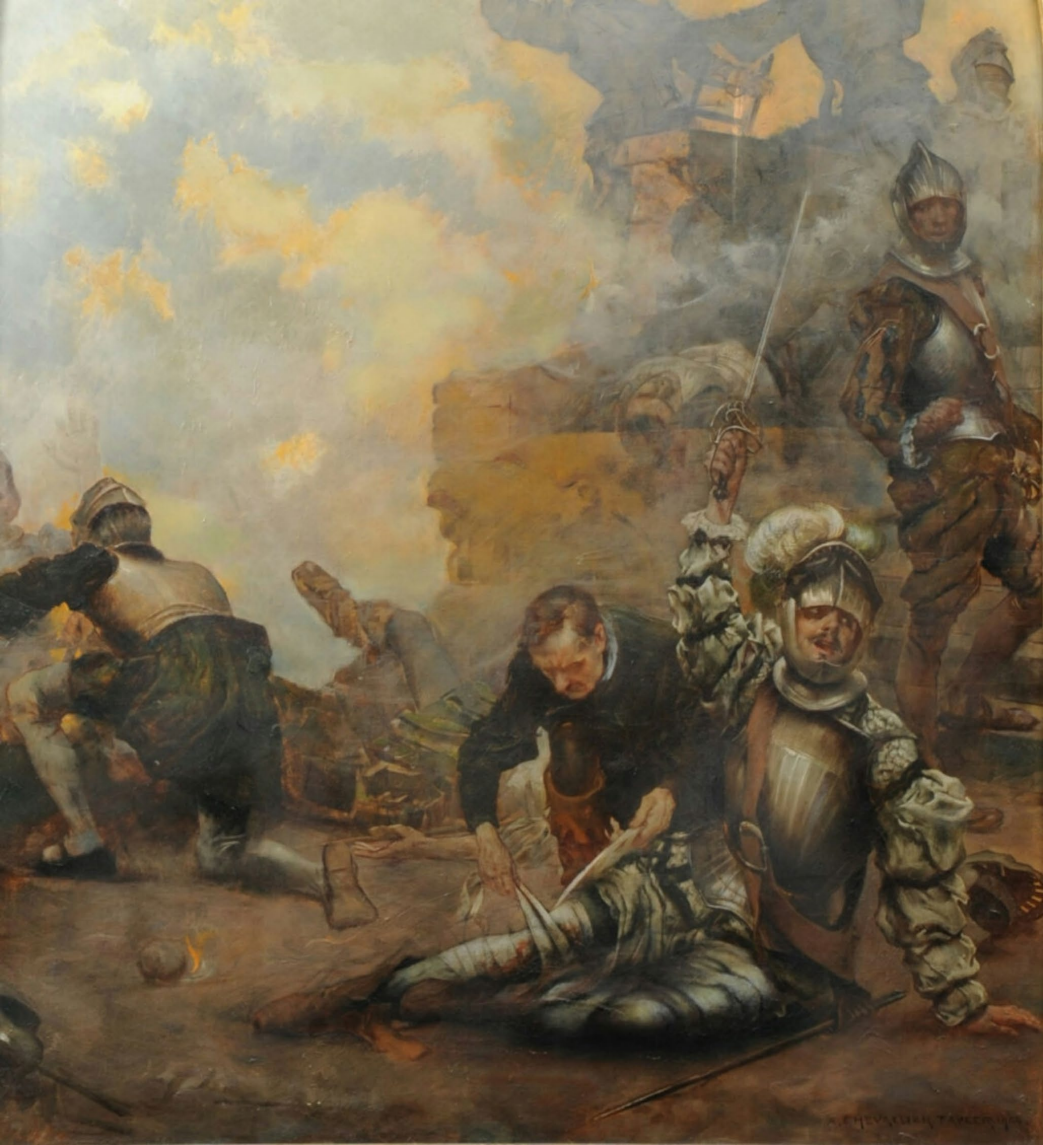
Painting: “Ignatius falls in Pamplona” (Albert Chevallier-Tayler − 1904)

In May 1521, during the siege of Pamplona, Íñigo López, a knight serving the Spanish crown, suffered a catastrophic injury when a cannonball struck his leg “breaking it all” (Fig. 1). The injury resulted in a comminuted open fracture of the tibia. This type of injury, involving significant soft tissue damage and bone fragmentation, was frequently managed by amputation in that era due to the high risk of infection. However, due to the compassionate medical treatment by the French forces who captured the fortress, Ignatius was spared, his fracture was stabilized and he was transported to his castle.

Upon arrival, his condition worsened, and physicians and surgeons were summoned. They diagnosed a malposition of the bones, indicating that the initial reduction attempts were either poorly performed or that the fracture had displaced during transport. To address this, a drastic measure was taken: “to set the bones back in place, it was necessary to break the leg again.” Remarkably, Ignatius endured this excruciating intervention without a single complaint, only clenching his fists. This first documented non-union site revision surgery, likely an attempt of reduction of a displaced non-united fracture, was performed without the benefits of modern anaesthesia, which at the time was limited to rudimentary herbal substances like opium.

*“And*,* the day having come when the attack was expected*,* he made his confession with one of those companions of his in arms. And*,* after the attack had lasted a good time*,* a shot hit him in one leg*,* completely shattering it for him; and because the ball passed between both legs*,* the other was badly wounded too. And so*,* with him falling*,* those in the stronghold then gave themselves up to the French.*

*These*,* having taken possession of the fortress*,* treated the wounded man very well*,* treating him courteously and in a friendly way. And after he had been twelve or fifteen days in Pamplona*,* they carried him on a litter to his home country. There*,* with him being in a very bad state and calling doctors and surgeons from many quarters*,* they judged that the leg had to be pulled apart again and the bones set in their places again*,* saying that*,* because they had been badly set on the other occasion or because they had become dislocated on the journey*,* they were out of place and in this state it couldn’t heal. And this butchery was done again*,* during which*,* just as during all the others he had previously undergone and later underwent*,* he never spoke a word*,* nor showed any sign of pain other than clenching his fists tightly.”* [1].

### The persistent deformity and the second surgery

Despite the initial revision, the fracture consolidated with a significant residual deformity. Below the knee, one bone remained overlapped, resulting in a shortened leg, and a prominent bony protrusion was also present. This disfigurement was unacceptable to Ignatius, who, at the time, still intended to pursue a worldly life and found the defect “unbecoming”. He inquired of his physicians whether the protruding bone could be removed. The doctors cautioned that the pain would be far worse than anything he had experienced, due to the bone being already healed and the length of the procedure.

Nonetheless, driven by a strong will, Ignatius decided to undergo this “martyrdom”. His elder brother was reportedly terrified at the prospect, but Ignatius faced it with his characteristic fortitude. The procedure involved incising the flesh and sawing off the protruding bone. This clearly describes an osteotomy performed to correct the deformity. To address the shortening and prevent it from worsening, the physicians employed various remedies, including “various unguents” and, notably, “continuous traction”. This prolonged traction, lasting “days and days of martyrdom,” strongly suggests an attempt to correct both angular deformity and achieve some degree of limb lengthening. Pietro Panzeri, a key figure in Italian orthopaedics in the late 19th century, cited this case as the first known instance of such an operation in his 1881 publication “Annotazioni di chirurgia ortomorfica” [8].

*“And as the bones were at this point coming to knit one with another*,* he was left with one bone above his knee mounted on top of the other. Thus the leg was left shorter and the bone at that point protruded so much as to be something ugly. As he could not bear this (for he was set on following the world and he considered this would disfigure him)*,* he found out from the surgeons whether it could be cut. They said that it certainly could be cut*,* but that the pain would be greater than all those he had undergone before*,* given it was now healed and it would need time to cut it. And still he decided to make a martyr of himself out of self-will*,* though his elder brother was horrified and was saying that such pain he himself wouldn’t dare suffer. The injured man suffered it with his usual forbearance. And once the flesh and the excess bone at that point had been cut*,* the concern was to use remedies whereby the leg would not be left so short*,* applying many ointments to it and stretching it continually with appliances*,* which on many days were making a martyr of him.”* [1].

### The spiritual transformation

**Fig. 2:**
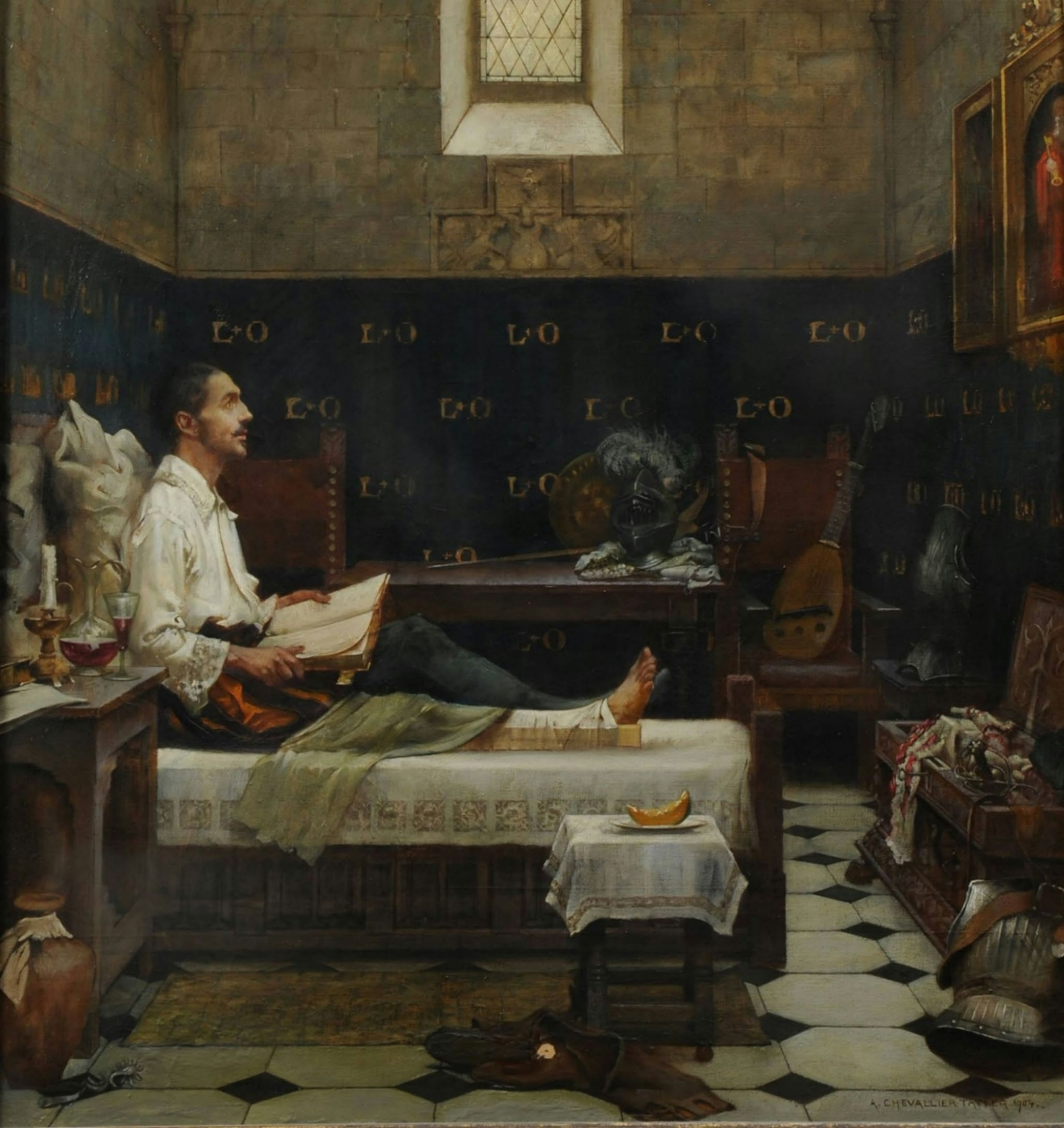
Painting “Ignatius convalesces at Loyola” (Albert Chevallier-Tayler − 1904)

Beyond the physical ordeal, Ignatius’s prolonged convalescence and intense suffering proved to be a profound turning point in his life. Confined to bed, he began reading religious texts, including “The Life of Christ” and “Flowers of the Saints,” having exhausted his preferred chivalric romances (Fig. 2).

**Fig. 3:**
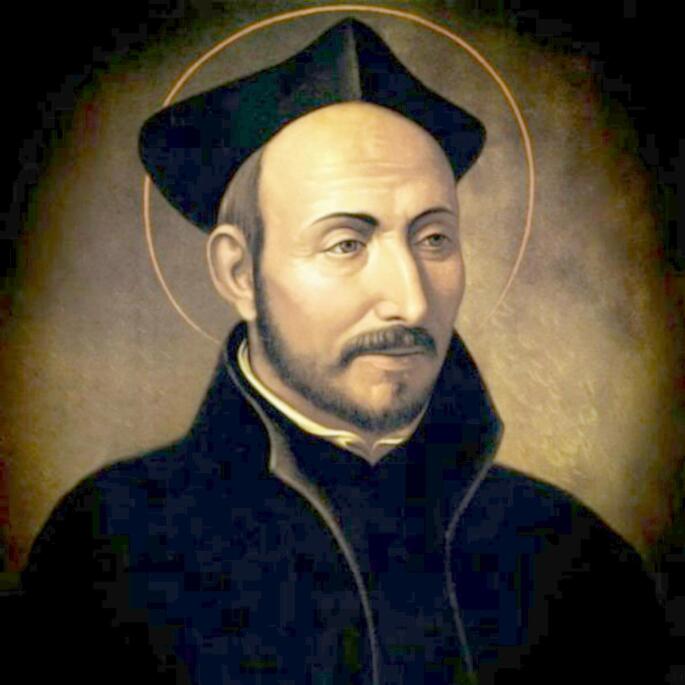
Portrait of St. Ignatius (Jacopino del Conte − 1556)

This period of enforced idleness and spiritual contemplation led to a profound conversion experience. The physical suffering he endured, far from breaking his spirit, became an integral part of his spiritual journey, moving him away from his former worldly ambitions towards a deep devotion to God. This transformative experience ultimately led him to found the Society of Jesus (the Jesuits), one of the most influential religious orders in history. This treatment not only represents a significant, early chapter in orthopaedic history but also marked the beginning of a life dedicated to spiritual service, forever altering the course of religious history (Fig. 3).

*“But Our Lord was gradually giving him health*,* and he was in such a good state that he was cured in all other respects except that he could not hold himself well on his leg*,* and thus he was forced to be in bed.*

*And because he was much given to reading worldly and false books*,* which they normally call ‘tales of chivalry’*,* he asked*,* once he was feeling well*,* that they give him some of these to pass the time. But in that house none of those books which he normally read could be found*,* and so they gave him a life of Christ and a book of the lives of the saints in Spanish.”* [1].

### Functional recovery and residual symptoms

Crucially, despite the severity of the injury and the rudimentary nature of the treatments, Ignatius’s recovery allowed him to regain significant functional independence. He was able to walk again, though he may have limped. More remarkably, he was able to ride a horse, an essential mode of transport at the time. The only notable lasting symptom was a chronic swelling in his leg by the evening, which necessitated replacing one shoe with a bandage. This demonstrates a remarkable level of recovery for such a complex injury treated in the 16th century.

*“And he also bought some canvas sandals*,* of which he wore just one. And this not for decorum’s sake but because he had one of his legs all tied up in a bandage and in a rather bad state: so much so that*,* although he was travelling on horseback*,* each night he found it swollen. This foot he felt he had to have shod.”* [1].

### Renaissance surgical advances and contemporaneous practices: Hans von gersdorff’s ‘fieldbook of surgery’

**Fig. 4:**
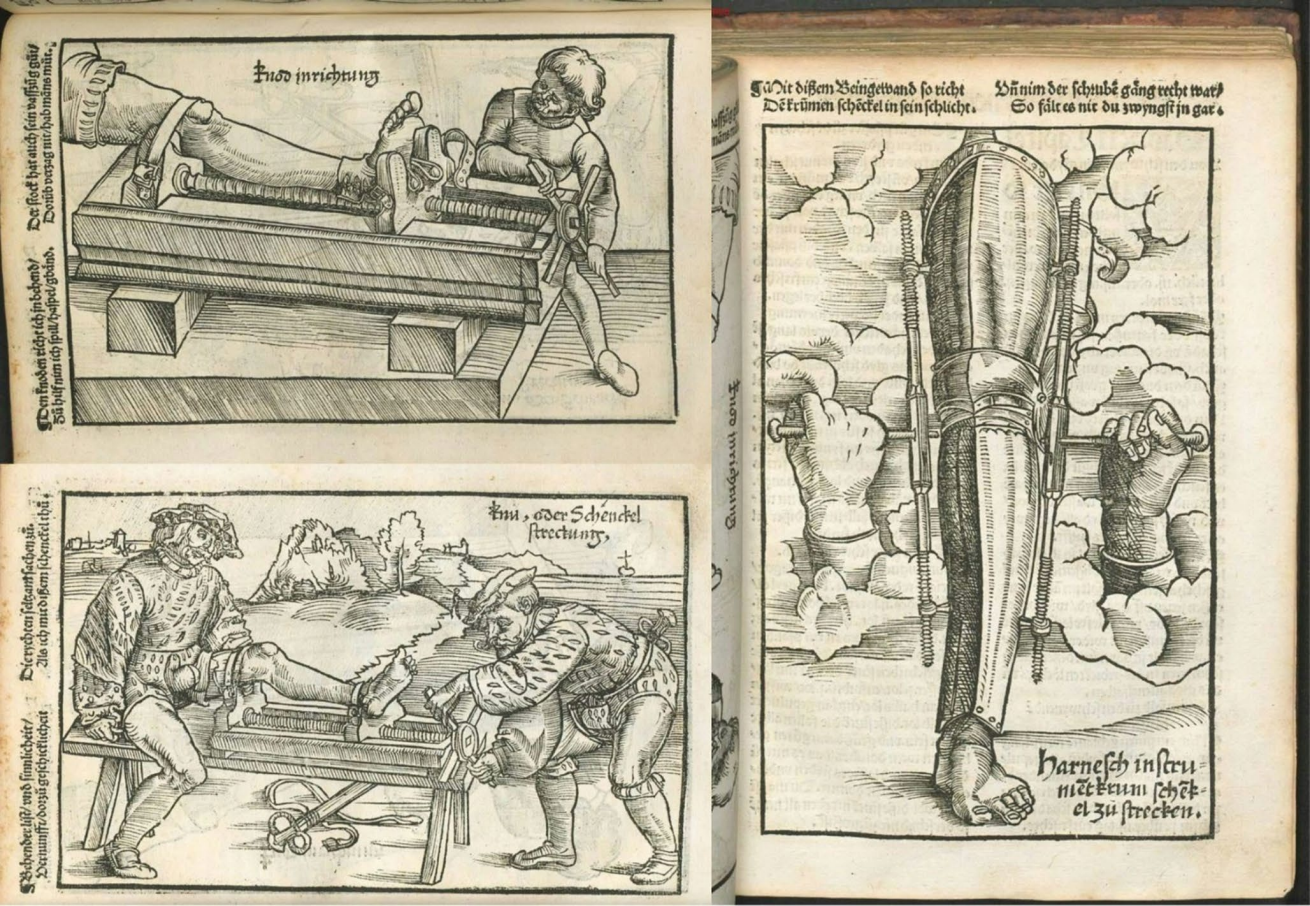
Illustrations from Hans von Gersdorff’s ‘Fieldbook of Surgery’

The medical care received by St. Ignatius of Loyola, particularly the complex sequence of procedures, serves as a compelling testament to the remarkable, albeit often overlooked, surgical progress achieved during the Renaissance. This period, spanning from the 14th to the 17th century, was characterized by a fervent revival of classical learning and a flourishing of new ideas across all fields, including medicine, anatomy, and technology [9]. The ability of surgeons to attempt such intricate bone procedures, even with rudimentary methods and without modern asepsis or anesthesia, reflects a growing practical skill and a willingness to push the boundaries of what was considered surgically possible. Simultaneously, at almost the same time as Ignatius’s surgeries, significant surgical knowledge was being compiled and disseminated elsewhere in Europe [10]. In 1517, just a few years before Ignatius’s injury, the German surgeon Hans von Gersdorff published his influential work, Feldbůch der Wundartzney, or “Fieldbook of Surgery” [11]. This comprehensive text, aimed primarily at military surgeons, detailed various surgical techniques for managing war wounds, fractures, amputations, and other conditions prevalent on the battlefield. Gersdorff’s work, illustrated with woodcuts shown in Fig. 4, provided practical guidance on bandaging, wound care, and the use of surgical instruments [11]. While it mentioned traction only for realignment of fractures and did not specifically detail limb lengthening procedures as performed on Ignatius, its existence underscores the active, albeit rudimentary, surgical scene in early 16th-century Europe, where military injuries were a primary driver for surgical innovation and the codification of existing practices [10]. Ignatius’s case, therefore, stands as a prime example of the often-astonishing capabilities reached by medical practitioners in an era that laid crucial groundwork for future scientific and technological breakthroughs.

## Conclusion

The case of St. Ignatius of Loyola, occurring in the early 16th century, offers a remarkable glimpse into early orthopaedic practices. The management of his comminuted, open tibial fracture involved not only a revision of the non-union site but also a subsequent osteotomy for deformity correction, combined with what appears to be a rudimentary form of continuous traction for limb lengthening. This predates many formally recognized developments in orthopaedic surgery and highlights the ingenuity and perseverance of both patients and surgeons in an era of limited medical knowledge and technology. While the tools and understanding were primitive, the underlying principles– anatomical reduction, stabilization, deformity correction, and attempts at lengthening– resonate with modern orthopaedic concepts. Surgical procedures performed on St. Ignatius’s can thus be considered a pioneering, albeit painful, precursor to today’s sophisticated limb lengthening and reconstruction procedures, and importantly, illustrates the profound impact of physical trauma and its treatment on an individual’s life trajectory, leading to a significant spiritual conversion.

## Data Availability

No datasets were generated or analysed during the current study.
